# Drug screening in human physiologic medium identifies uric acid as an inhibitor of rigosertib efficacy

**DOI:** 10.1172/jci.insight.174329

**Published:** 2024-05-30

**Authors:** Vipin Rawat, Patrick DeLear, Prarthana Prashanth, Mete Emir Ozgurses, Anteneh Tebeje, Philippa A. Burns, Kelly O. Conger, Christopher Solís, Yasir Hasnain, Anna Novikova, Jennifer E. Endress, Paloma González-Sánchez, Wentao Dong, Greg Stephanopoulos, Gina M. DeNicola, Isaac S. Harris, David Sept, Frank M. Mason, Jonathan L. Coloff

**Affiliations:** 1 Department of Physiology and Biophysics, University of Illinois College of Medicine, University of Illinois Cancer Center, Chicago, Illinois, USA.; 2 Department of Biomedical Engineering, University of Michigan, Ann Arbor, Michigan, USA.; 3 Division of Hematology and Oncology, Department of Medicine, Vanderbilt University Medical Center, Nashville, Tennessee, USA.; 4 Department of Nutrition and Integrative Physiology, Florida State University, Tallahassee, Florida, USA.; 5 Meyer Cancer Center, Weill Cornell Medicine, New York, New York, USA.; 6 Department of Metabolism and Physiology, Moffitt Cancer Center, Tampa, Florida, USA.; 7 Department of Chemical Engineering, Massachusetts Institute of Technology, Cambridge, Massachusetts, USA.; 8 Department of Biomedical Genetics, Wilmot Cancer Institute, University of Rochester Medical Center, Rochester, New York, USA.

**Keywords:** Cell biology, Oncology, Cancer, Cytoskeleton, Drug screens

## Abstract

The nonphysiological nutrient levels found in traditional culture media have been shown to affect numerous aspects of cancer cell physiology, including how cells respond to certain therapeutic agents. Here, we comprehensively evaluated how physiological nutrient levels affect therapeutic response by performing drug screening in human plasma-like medium. We observed dramatic nutrient-dependent changes in sensitivity to a variety of FDA-approved and clinically trialed compounds, including rigosertib, an experimental cancer therapeutic that recently failed in phase III clinical trials. Mechanistically, we found that the ability of rigosertib to destabilize microtubules is strongly inhibited by the purine metabolism end product uric acid, which is uniquely abundant in humans relative to traditional in vitro and in vivo cancer models. These results demonstrate the broad and dramatic effects nutrient levels can have on drug response and how incorporation of human-specific physiological nutrient medium might help identify compounds whose efficacy could be influenced in humans.

## Abstract 2

Uric acid inhibits the efficacy of the microtubule-destabilizing agent rigosertib against cancer cells.

## Introduction

Tumor growth is influenced by both cell-intrinsic and cell-extrinsic factors, and nutrient availability is emerging as a critical environmental factor that can shape the metabolic fitness and proliferative capacity of tumors (1–3). Concurrent with these discoveries has been a renewed realization that standard cell culture media were not designed to mimic the nutrient environment found in vivo but were designed to provide excess amounts of the minimal nutrients required to sustain cancer cell growth in vitro (4–9). As a result, the nutrients present in these traditional culture media do not accurately recapitulate the complexity or abundance of nutrients found in vivo. Importantly, it has recently been shown that the highly nonphysiological nutrient levels found in culture media can contribute to inconsistencies between in vitro and in vivo experiments, especially for those directly related to cellular metabolism (10–13). It has also been observed that nutrient availability can affect the response to a variety of cancer therapies (14), including traditional chemotherapies (15, 16), metabolic inhibitors (17, 18), targeted therapies (19, 20), and immunomodulatory checkpoint inhibitors (21).

Because of the importance of nutrient availability in influencing tumor metabolic phenotypes and therapeutic vulnerabilities, there is considerable interest in targeting systemic metabolism either alone or in combination with existing therapies to treat cancer. This includes several dietary interventions that are being investigated as components of cancer therapies (22, 23), including amino acid starvation (24–26), ketogenic diet (27, 28), caloric restriction (29–31), and fasting-mimicking diets (32). The success of dietary intervention studies is critically dependent on mouse models and has led to tremendous interest in translating these findings to patients. Although mouse models provide an essential platform to study interactions between systemic and tumor metabolism, a number of metabolic differences between mice and humans influence tumor biology (33–36), including how cancer cells respond to cancer therapeutics (15). These issues have motivated the development of novel culture media that specifically mimic the nutrient composition found in human plasma as platforms for studying therapeutic response under more physiological human nutrient conditions (12, 15).

Here, we sought to determine the extent to which nutrient availability affects the sensitivity of cancer cells to diverse therapeutic agents by using a high-throughput, differential-sensitivity drug-screening platform to profile therapeutic sensitivity in cancer cells growing in traditional versus physiological human plasma-like medium (HPLM). This screen revealed dramatic nutrient-dependent changes in sensitivity to a wide variety of drugs in cells cultured in HPLM. Among these differences were changes in sensitivity to the experimental therapeutic rigosertib (ON-01910), the efficacy of which was strongly antagonized by the purine degradation product uric acid.

## Results

### Drug screening identifies nutrient-dependent effects on drug response.

**Figure 1:**
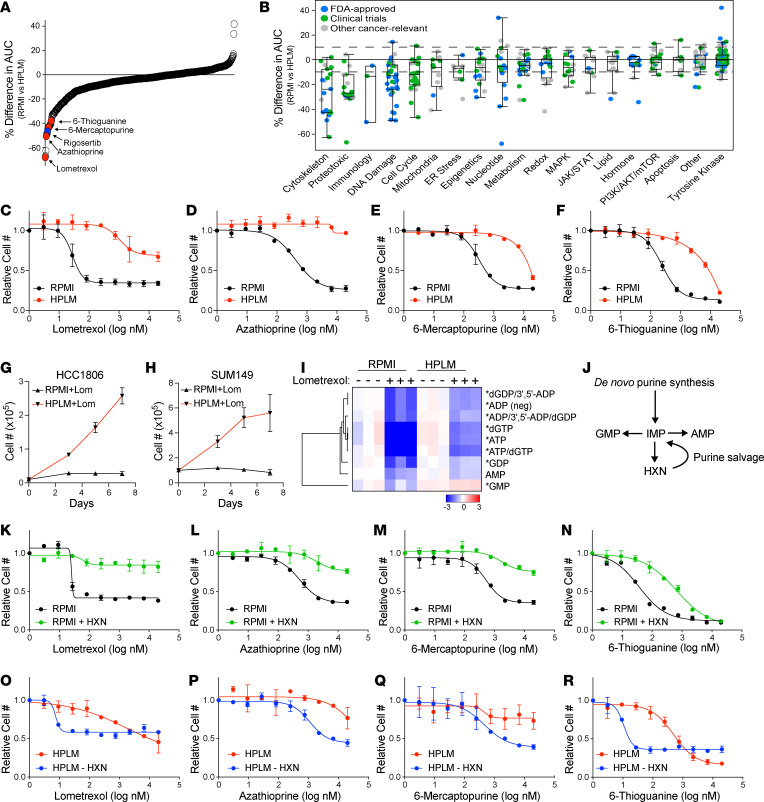
Culture in HPLM changes sensitivity to a variety of therapeutic agents. (**A**) Percentage difference in the area under curve (% difference in AUC) data for SUM149 cells cultured in either RPMI or HPLM after treatment with anticancer and metabolic inhibitor libraries. Only compounds with a maximum effect of more than 50% in either medium are shown. (**B**) The same data as in **A** categorized based on target pathway. Box plots show the interquartile range, median (line), and minimum and maximum (whiskers). (**C**–**F**) Dose-response curves of the purine biosynthesis inhibitors lometrexol (**C**), azathioprine (**D**), 6-mercaptopurine (**E**), and 6-thioguanine (**F**) on SUM149 cells growing in RPMI versus HPLM. (**G** and **H**) Growth curves of HCC1806 (**G**) and SUM149 (**H**) cells treated with lometrexol in RPMI versus HPLM. (**I**) LC-MS analysis to quantify purine nucleotide abundance in HCC1806 cells treated with lometrexol in RPMI versus HPLM. * indicates *P* < 0.05 for HPLM + lometrexol relative to RPMI + lometrexol (unpaired 2-tailed *t* test). (**J**) Schematic representation of purine synthesis and salvage pathways. (**K**–**N**) Dose-response curves of the purine biosynthesis inhibitors lometrexol (**K**), azathioprine (**L**), 6-mercaptopurine (**M**), and 6-thioguanine (**N**) on SUM149 cells grown in RPMI with and without hypoxanthine (HXN). (**O**–**R**) Dose-response curves of the purine biosynthesis inhibitors lometrexol (**O**), azathioprine (**P**), 6-mercaptopurine (**Q**), and 6-thioguanine (**R**) on SUM149 cells grown in HPLM with and without HXN. For all panels data represent the means ± SD of triplicate samples.

Commercial culture media, such as DMEM and RPMI-1640 (RPMI), contain nutrients at nonphysiological levels and lack many critical components present in human plasma (37). Due to these deficiencies, several labs have recently developed media that more accurately mimic physiological nutrient levels found in human circulation (10–12, 15). We have made use of HPLM (15) to culture breast cancer cell lines, in which after a 2-week adaptation period we observed similar or slightly decreased growth rates (Supplemental Figure 1A; supplemental material available online with this article; https://doi.org/10.1172/jci.insight.174329DS1) and consistent remodeling of intracellular metabolite abundance (Supplemental Figure 1, B and C). Because of the recent observation of the impact of nutrient availability and cellular metabolism on the response to a variety of cancer therapies (15, 16, 20), we hypothesized that culturing cancer cells in HPLM would change how they respond to therapeutic agents on a larger scale. To address this hypothesis, we used a high-throughput, differential-sensitivity drug-screening platform containing a library of 626 metabolic inhibitors and anticancer compounds arrayed in 10-point dose curves (38). This platform contains compounds targeting a wide variety of cancer-relevant pathways, many of which are FDA approved or have been evaluated in clinical trials. We screened the triple-negative breast cancer (TNBC) cell line SUM149 growing in RPMI or HPLM, where we observed dramatic changes in sensitivity to a variety of compounds (Figure 1, A and B, and Supplemental Table 1). Interestingly, while very few drugs were more effective in HPLM, a large proportion of drugs were less effective in physiological medium.

Among the drugs most strongly affected by culture in HPLM are 4 inhibitors of the de novo purine biosynthesis pathway — lometrexol, azathioprine, 6-thioguanine, and 6-mercaptopurine — all of which are less effective at reducing cell numbers in HPLM (Figure 1, A–F). We found that both SUM149 cells and another TNBC cell line, HCC1806, are able to proliferate when treated with lometrexol in HPLM but not RPMI (Figure 1, G and H). Based on these observations, we investigated the level of purine nucleotides by liquid chromatography–mass spectrometry (LC-MS) analysis in HCC1806 cells treated with and without lometrexol in both RPMI and HPLM. As expected, we found that lometrexol caused a large drop in the abundance of most purine nucleotides in RPMI; however, this drop was significantly blunted in HPLM (Figure 1I). In addition to de novo biosynthesis, cells can acquire purines through the purine salvage pathway (Figure 1J), and the presence of substrates for the purine salvage pathway, such as hypoxanthine, reduces the efficacy of purine synthesis inhibitors (39, 40). Although traditional media formulations do not contain salvage substrates, HPLM contains hypoxanthine at 10 μM as is found in human plasma. Indeed, we found that addition of hypoxanthine to RPMI was sufficient to provide resistance against these compounds (Figure 1, K–N), and the removal of hypoxanthine from HPLM strongly increased the sensitivity of SUM149 cells to purine biosynthesis inhibitors (Figure 1, O–R). While the ability of hypoxanthine to rescue purine synthesis inhibitors is known, these results demonstrate the power and utility of our screening platform to identify physiological nutrients that modify cancer cell sensitivity to therapeutic agents.

### Uric acid in HPLM reduces cancer cell sensitivity to rigosertib in vitro.

**Figure 2:**
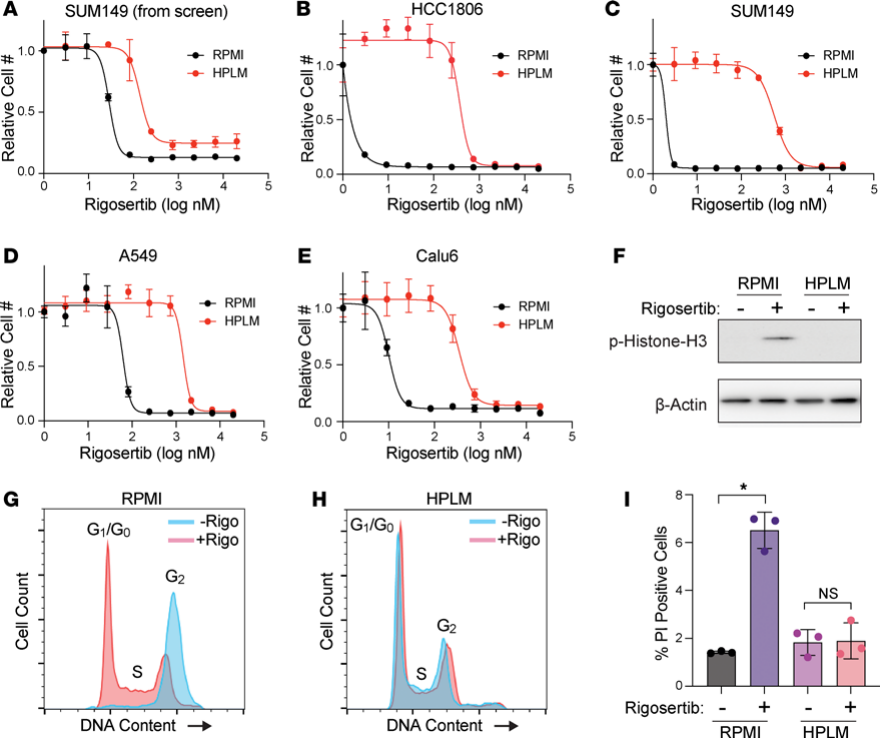
Culture in HPLM reduces sensitivity to rigosertib. (**A**) Dose-response curve of SUM149 cells treated with rigosertib from the high-throughput screen described in Figure 1. Data are the mean ± SD of triplicate samples. (**B**–**E**) Dose-response curves for rigosertib treatment of HCC1806 (**B**), SUM149 (**C**), A549 (**D**), and Calu6 (**E**) cells growing in RPMI versus HPLM. Data are the mean ± SD of triplicate samples. (**F**) Representative Western blot of phosphorylated histone H3 in HCC1806 cells treated with 150 nM rigosertib in RPMI versus HPLM. (**G** and **H**) Cell cycle analysis of HCC1806 cells treated with 150 nM commercial-grade rigosertib in RPMI (**G**) and HPLM (**H**). (**I**) Cell death analysis of HCC1806 cells treated with 200 nM commercial-grade rigosertib in RPMI versus HPLM. PI, propidium iodide. Cell death and cell cycle data are the means ± SD of triplicate samples. * indicates *P* < 0.05 by unpaired 2-tailed *t* test.

Another top hit from our screen was the experimental cancer therapeutic rigosertib, which was markedly less effective against cells growing in HPLM than in RPMI (Figure 1A and Figure 2A). We validated these results by performing rigosertib dose-response analyses in HCC1806 and SUM149 cells, where we observed 2,711- and 283-fold increases (respectively) in the IC_50_ for rigosertib in HPLM (Figure 2, B and C). Similar results were obtained in 2 lung cancer cell lines, A549 and Calu6, suggesting that this effect is likely general and not restricted to breast cancer cells (Figure 2, D and E). Rigosertib’s anticancer effects have been shown to be mediated by induction of both G2/M cell cycle arrest and cell death (41, 42). Accordingly, we treated the HCC1806 cells with 150 nM rigosertib, a dose we identified to reduce cell number only in RPMI (Figure 2B), and observed that rigosertib induced phosphorylation of histone H3 and G2/M cell cycle arrest in RPMI but not in HPLM. Similarly, induction of cell death by treating HCC1806 cells with 200 nM rigosertib was blocked in HPLM (Figure 2, F–I).

**Figure 3:**
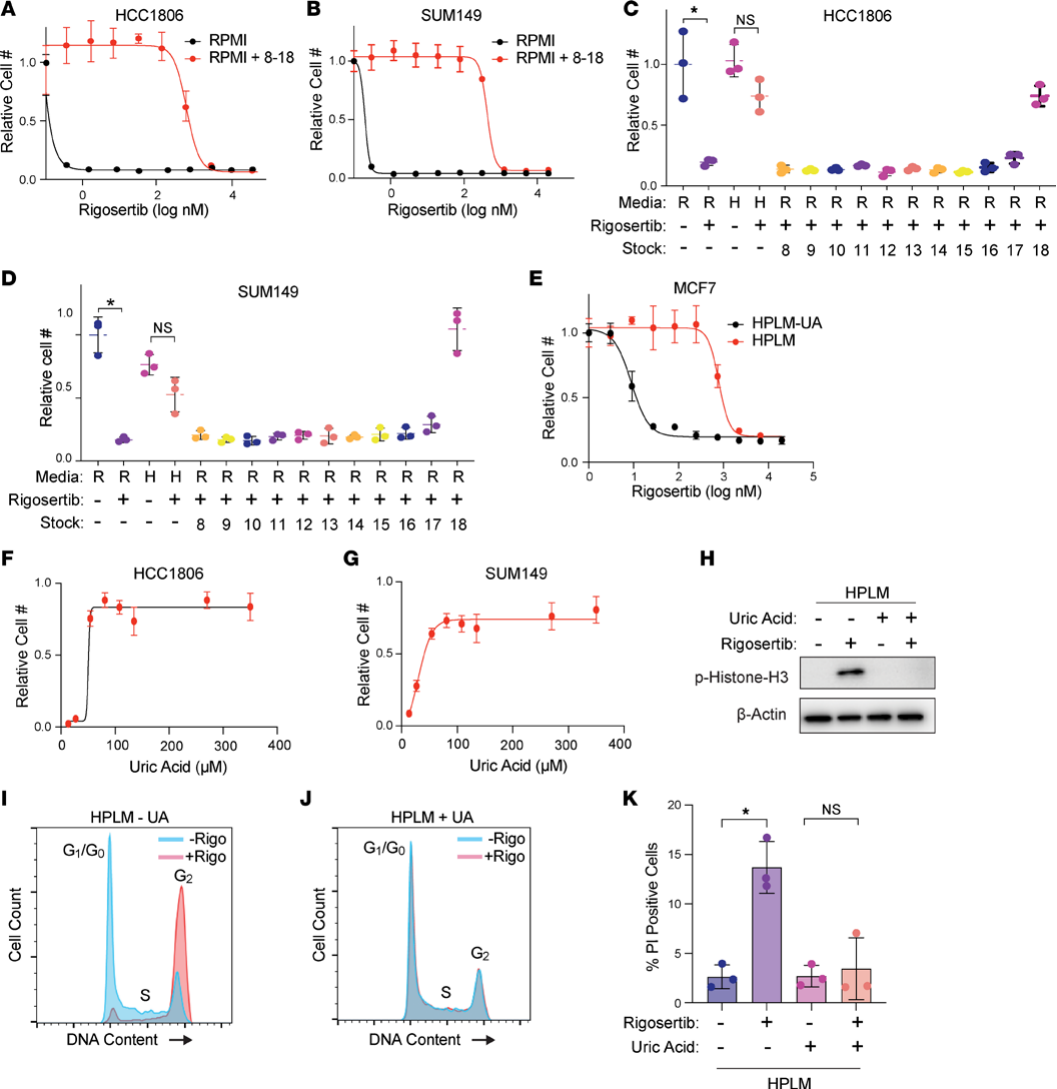
Uric acid prevents the activity of rigosertib. (**A** and **B**) Dose-response curves of HCC1806 (**A**) and SUM149 (**B**) cells treated with rigosertib in RPMI versus RPMI + HPLM stocks 8–18. (**C** and **D**) Cell growth assays of HCC1806 (**C**) and SUM149 (**D**) cells treated with 80 nM rigosertib in the presence of individual HPLM stocks 8–18. R, RPMI; H, HPLM. (**E**) Dose-response curve of MCF7 cells treated with rigosertib in HPLM versus HPLM – UA. UA, uric acid. (**F** and **G**) Dose-response curves of uric acid on HCC1806 (**F**) and SUM149 (**G**) cells treated with 80 nM rigosertib. (**H**) Representative Western blot of phosphorylated histone H3 in HCC1806 cells treated with 150 nM rigosertib in HPLM versus HPLM – UA. (**I** and **J**) Cell cycle analysis of HCC1806 cells treated with 150 nM commercial-grade rigosertib in HPLM (**I**) and HPLM – UA (**J**). (**K**) Cell death analysis of HCC1806 cells treated with 200 nM commercial-grade rigosertib in HPLM and HPLM – UA. For all panels, data are represented as mean ± SD of triplicate samples. * indicates *P* < 0.05 by unpaired 2-tailed *t* test.

Next, we sought to determine the component(s) of HPLM that antagonizes rigosertib activity. Like RPMI and other traditional media, HPLM consists of glucose, amino acids, and salts, albeit at different concentrations (15). HPLM contains 27 additional components not found in RPMI but found in human plasma. Most of these unique ingredients are organized into 11 stock solutions numbered 8 through 18. To determine whether a unique component of HPLM is responsible for the reduced sensitivity to rigosertib, we combined HPLM stocks 8–18, added them to RPMI, and performed dose-curve analyses, in which we found that stocks 8–18 were able to recapitulate the effect of HPLM on rigosertib sensitivity (Figure 3, A and B). We then analyzed stocks 8–18 individually and found that addition of stock 18 alone was sufficient to protect against rigosertib in RPMI (Figure 3, C and D). Importantly, stock 18 contains only 1 component: the purine metabolism waste product uric acid, which is present in human plasma and HPLM at 350 μM. Indeed, we found that removal of uric acid from HPLM was sufficient to dramatically increase cancer cell sensitivity to rigosertib (Figure 3E). We verified the broad protective effects of uric acid by creating rigosertib dose curves on multiple cancer cell lines of different origin, including lung, renal, and chronic myeloid leukemia, where we observed the protective effects of uric acid in all cases (Supplemental Figure 2). To determine whether uric acid protects cells from rigosertib in a dose-dependent manner, we created a dose curve of uric acid in RPMI in the presence of 80 nM rigosertib. Interestingly, we found that uric acid concentrations as low as 27 μM were able to partially protect against rigosertib (Figure 3, F and G). Similar to HPLM, physiological concentrations of uric acid alone were sufficient to block the ability of 150 nM rigosertib from inducing histone H3 phosphorylation and G2/M cell cycle arrest. Similarly, induction of cell death by treatment of HCC1806 cells with 200 nM rigosertib was blocked in the presence of uric acid (Figure 3, H–K).

### Uric acid inhibits the microtubule-destabilizing activity of pharmaceutical-grade rigosertib.

**Figure 4:**
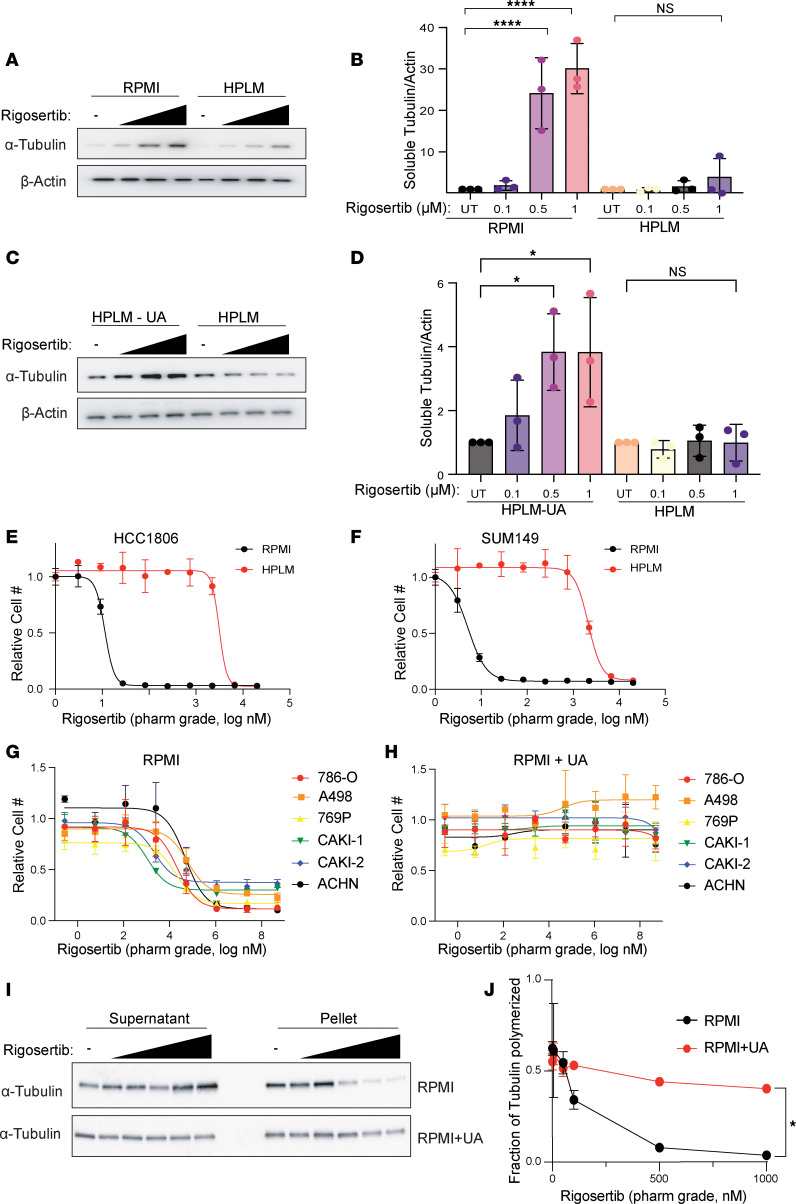
Uric acid inhibits the microtubule-destabilizing activity of rigosertib. (**A**) Western blot of soluble α-tubulin from SUM149 cells treated with increasing doses of rigosertib (0.1 μM, 0.5 μM, and 1 μM) for 4 hours in RPMI and HPLM. (**B**) Quantification of Western blots from **A**. Data are represented as mean ± SD from 3 independent experiments. ****P < 0.0001, *P < 0.05 by 1-way ANOVA followed by Tukey’s multiple-comparison test. (**C**) Western blot of soluble α-tubulin from SUM149 treated with increasing doses of rigosertib (0.1 μM, 0.5 μM, and 1 μM) for 4 hours in HPLM and HPLM – UA. (**D**) Quantification of Western blots from **C**. Data are represented as mean ± SD from 3 independent experiments. *P < 0.05 by 1-way ANOVA followed by Tukey’s multiple-comparison test. (**E** and **F**) Dose-response curves of HCC1806 (**E**) and SUM149 (**F**) cells treated with pharmaceutical-grade rigosertib in RPMI versus HPLM. (**G** and **H**) Dose-response curves of a panel of renal cancer cell lines treated with pharmaceutical-grade rigosertib in RPMI (**G**) versus RPMI + UA (**H**). (**I**) Western blot of soluble and pellet α-tubulin from 786-O cells treated with increasing doses (5 nM, 50 nM, 100 nM, 500 nM, 1,000 nM) of pharmaceutical-grade rigosertib for 4 hours in RPMI and RPMI + UA. (**J**) Quantification of Western blots from **I**. Data are represented as means ± SD of 3 independent experiments. *P < 0.05 by 2-way ANOVA.

While the mechanism of action of rigosertib remains controversial (43–46), several recent reports have demonstrated that rigosertib is a microtubule-destabilizing agent that binds tubulin dimers at the colchicine binding site (41, 47, 48). To verify the ability of rigosertib to destabilize microtubules, we performed short-term treatments (4 hours) of HCC1806 and SUM149 cells cultured in RPMI with increasing doses of commercial-grade rigosertib, where we observed increased levels of α-tubulin in the soluble fraction of cell lysates, suggesting that rigosertib does indeed destabilize microtubules (Figure 4, A and B, and Supplemental Figure 3, A and B). Importantly, however, the ability of rigosertib to destabilize microtubules in cells grown in HPLM was strongly inhibited (Figure 4, A and B, and Supplemental Figure 3, A and B). To determine whether uric acid prevents rigosertib-mediated microtubule destabilization, we treated cells cultured in HPLM with and without 350 μM uric acid with commercial-grade rigosertib, which resulted in a dose-dependent increase in the level of soluble α-tubulin only in the absence of uric acid (Figure 4, C and D, and Supplemental Figure 3, C and D).

Previous work has shown that the presence of a contaminant in commercial-grade rigosertib may contribute to its anticancer effects (49). To determine whether HPLM blocks the effect of rigosertib or a potential contaminant, we made use of pharmaceutical-grade rigosertib that lacks the potentially active contaminant. Similar to commercial-grade rigosertib, culture of cells in HPLM strongly reduced the cellular sensitivity to pharmaceutical-grade rigosertib (Figure 4, E and F). Importantly, addition of 350 μM uric acid to RPMI prevented sensitivity to pharmaceutical-grade rigosertib in a panel of renal cancer cell lines (Figure 4, G and H), indicating that uric acid is protective against rigosertib and not a contaminant found in the commercial-grade compound. Similarly, treatment of 786-O cells with pharmaceutical-grade rigosertib resulted in a dose-dependent decrease in α-tubulin found in the pellet (microtubule fraction) when compared with total tubulin in RPMI, but the addition of uric acid to RPMI prevented rigosertib-mediated destabilization of microtubules (Figure 4, I and J).

### Uric acid may weaken the rigosertib/β-tubulin interaction.

**Figure 5:**
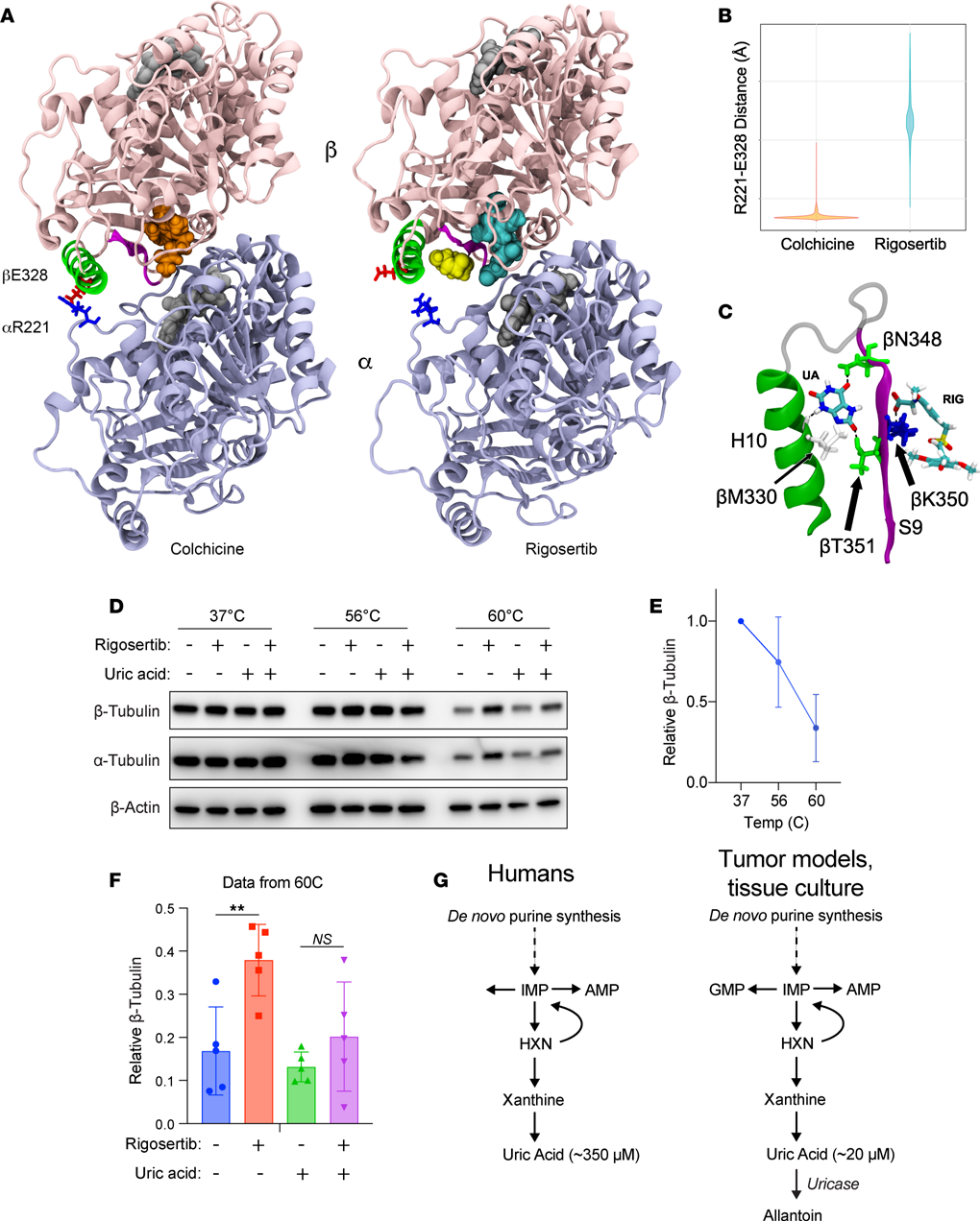
Uric acid inhibits rigosertib activity by reducing the affinity of rigosertib for β-tubulin. (**A**) Structural comparisons of colchicine-bound and rigosertib-bound tubulin. Colchicine and rigosertib are colored orange and cyan, respectively. The salt bridge between βE328 and αR221 found in the colchicine structure is absent in the rigosertib structure, allowing H10 (shown in green) to move away from the dimer body and create a pocket for uric acid (shown in yellow) to bind. (**B**) Distance between βE328 and αR221 in the colchicine and rigosertib simulations. When this ionic bond is not formed, H10 becomes untethered, which creates the binding pocket for uric acid. (**C**) Molecular details of uric acid binding in the pocket between H10 (green) and S9 (magenta). Residues that form hydrogen bonds with uric acid are labeled. (**D**) CETSA analysis of K562 cells treated for 4 hours with 40 μM pharmaceutical-grade rigosertib in RPMI at the indicated temperature. (**E**) Quantification of β-tubulin melting at increasing temperature in the absence of uric acid and rigosertib. *N* = 5 independent experiments. (**F**) Quantification of β-tubulin at 60°C in the presence and absence of rigosertib and uric acid. Data are represented as means ± SD of 5 independent experiments. **P < 0.01 from unpaired 2-tailed *t* test. (**G**) Unlike mice and other model organisms and systems, humans do not express uricase, resulting in uniquely high uric acid levels.

The acute ability of uric acid to prevent rigosertib-mediated destabilization of microtubules motivated us to explore the potential molecular effects of rigosertib and uric acid on tubulin structure. As a benchmark, we compared rigosertib with colchicine in our analyses. We started by performing molecular dynamics (MD) simulations of colchicine-bound tubulin, rigosertib-bound tubulin, and apo-tubulin (non-drug bound control). After equilibrating each structure for 0.5 μs, we performed 4 independent simulations of each complex, resulting in more than 6 μs of total simulation time. Using principal component analysis to evaluate the large-scale structural differences induced by colchicine and rigosertib, we found that both colchicine and rigosertib produced a similar “kink” in the dimer that likely explains their ability to prevent microtubule polymerization (Supplemental Figure 4 and Supplemental Videos 1 and 2). In addition, rigosertib induced a conformational change that altered the relative orientation of α- and β-tubulin (Supplemental Figure 4 and Supplemental Videos 1 and 2). Specifically, the colchicine-bound simulation featured a persistent salt bridge formed between αR221 and βE328 that was directly adjacent to the colchicine binding site (Figure 5A). Since rigosertib altered the intradimer interface and created a greater distance between αR221 and βE328 (Figure 5B), this salt bridge cannot be formed in rigosertib-tubulin (Figure 5A). Helix H10 in β-tubulin contains E328, and loss of this salt bridge made H10 more dynamic and created a pocket between H10 and strand S9 (Figure 5C). Docking studies revealed that uric acid could bind within this pocket via hydrogen bonding with residues in both H10 and S9 (Figure 5C). Importantly, S9 also interacts with the carboxyl group of rigosertib, and using free energy calculations, we found that the likely effect of uric acid binding would be to weaken the binding affinity of rigosertib to β-tubulin. We evaluated this possibility using the cellular thermal shift assay (CETSA) (50), where we observed denaturation and precipitation of both α- and β-tubulin at 60°C that was strongly reduced in the presence of rigosertib, suggesting that rigosertib is capable of binding to tubulin (Figure 5, D–F), as has been shown by multiple other labs (41, 47, 48). However, addition of uric acid to the culture media significantly reduced the stabilization of α- and β-tubulin by rigosertib (Figure 5, D–F). These data are suggestive of a potential mechanism by which uric acid antagonizes rigosertib activity by weakening the interaction between rigosertib and β-tubulin, thereby acting as an uncompetitive inhibitor. However, additional studies will be required to determine whether uric acid directly interacts with rigosertib-bound β-tubulin or functions through other mechanisms.

## Discussion

Despite promising preclinical data and extensive evaluation in early-stage studies, rigosertib has thus far failed to improve outcome in the 2 phase III clinical trials in which it has been investigated (51, 52). Although there are likely numerous factors that have contributed to this poor clinical performance, our discovery that uric acid strongly antagonizes the microtubule-destabilizing activity of rigosertib in vitro suggests that the elevated levels of uric acid characteristic of humans may also contribute. Most species, including mice and others commonly used in cancer research (e.g., bovine serum), have a functional uricase gene that converts uric acid to the more soluble allantoin, resulting in relatively low circulating uric acid concentrations (Figure 5G). However, due to the evolutionarily recent pseudogenization of the uricase gene in humans and other closely related apes, humans have circulating uric acid levels that are an order of magnitude higher than other mammals (Figure 5G) (53–58). Further, patients with cancer, including those with myelodysplastic syndromes, where rigosertib has been most thoroughly investigated, often present with hyperuricemia (59, 60), and therefore, they may have uric acid levels that are even higher than those found in HPLM. Importantly, given that uric acid is the underlying cause of gout, there are numerous approved therapies to reduce uric acid levels in patients. Our work suggests that such therapies, including a low-purine diet, xanthine oxidase inhibitors (e.g., allopurinol, febuxostat), and uric acid–degrading enzymes (e.g., rasburicase, pegloticase), could be candidates to improve the therapeutic response to rigosertib (60–63).

As previously mentioned, identifying the precise mechanistic target of rigosertib has been challenging. Rigosertib was initially identified as a PLK1 inhibitor (41, 64–68) and has been proposed to act as an inhibitor of RAS (69) and PI3K (70). Use of an unbiased CRISPRi/a chemical-genetic approach combined with structural biology studies identified rigosertib as a microtubule-destabilizing agent that binds to the colchicine binding site on β-tubulin (47, 48), a finding that has now been corroborated by other groups (45). Our MD simulation studies also suggest that rigosertib binds to the colchicine binding site of β-tubulin and induces conformational changes that are similar to, but distinct from, those induced by colchicine. It is important to note, however, that the crystal structures of tubulin with colchicine or rigosertib are strongly affected by the presence of stathmin, and this limits the interpretation of these structures (47). In addition, docking results suggest that uric acid may act as an uncompetitive inhibitor of rigosertib through interaction with residues in loop S9 — a conclusion that is further verified by our CETSA results. Together, these results suggest that the effect of uric acid on rigosertib efficacy is mediated through microtubules and not the other proposed targets of rigosertib.

In vitro tissue culture models offer several advantages over in vivo tumor models, including the ability to perform large-scale screening studies. However, there has always been a large bottleneck of promising in vitro cancer findings that turn out to be irrelevant in human tumors. While there are many factors that contribute to this bottleneck, our work and that of others has shown that the nonphysiological nutrient levels found in traditional culture media likely contribute to some in vitro and in vivo discrepancies. Importantly, unnatural nutrient levels are not an inherent problem of tissue culture, and it is becoming clear that replacement of traditional media with more physiological media can rectify some of the problems with tissue culture systems. Although mouse models will continue to be the gold standard of preclinical cancer studies, it is important to consider the differences between mice and humans that could contribute to discrepancies in how cancer cells respond to therapies. Our work demonstrates that use of HPLM can lead to identification of drug/metabolite interactions that otherwise might be missed using traditional tissue culture or mouse models, and we support the notion that use of physiological media is a highly valuable addition to the cancer research pipeline.

## Methods

### Sex as a biological variable.

In this study we did not utilize any mice or human samples.

### Cell lines.

Cell lines were acquired from the Brugge Lab at Harvard Medical School, Boston, Massachusetts, USA (HCC1806, SUM149); the Kim Rathmell Lab at Vanderbilt University Medical Center, Nashville, Tennessee, USA (A498, 786-O, and Caki2); the Vadim Gaponenko Lab at the University of Illinois at Chicago, Chicago, Illinois, USA (K562); and the ATCC (A549 and Calu6). Cell lines were tested for mycoplasma using the MycoAlert Mycoplasma Detection Kit (Lonza) and were authenticated by single tandem repeat analysis. Cells were grown in HPLM according to the published formulation (15) with 5% dialyzed fetal bovine serum (FBS) (MilliporeSigma) and pen/strep (Invitrogen) at 37°C with 5% CO_2_. Medium was changed at least every 2 days. As needed, cells were incubated in RPMI (Thermo Fisher Scientific, 11875-093) with or without uric acid (MilliporeSigma) with 5% dialyzed FBS and pen/strep.

### Dose curve analysis.

To perform dose curve analysis, 2,000 cells were seeded in a 96-well plate in corresponding media. The next day cells were treated with the indicated compounds (lometrexol [MedChemExpress HY-14521], azathioprine [Selleckchem S1721], 6-mercaptopurine [Selleckchem S1305], 6-thioguanine [Selleckchem S1774], chemical-grade rigosertib [Selleckchem S1362], and pharmaceutical-grade rigosertib [Onconova Therapeutics]) by performing 9-point serial dilutions. The media were removed after 72 hours of drug treatment, and cells were fixed with 4% paraformaldehyde (MilliporeSigma F8775) for 10 minutes at room temperature. After fixation cells were washed with PBS and stained with 0.5 μg/mL Hoechst 33342 trihydrochloride (Thermo Fisher Scientific, H3570). Cell numbers were determined by imaging and quantifying nuclei using the Celigo imaging cytometer (Nexcelom).

### Growth curve analysis.

For growth curves 10,000 cells were plated in 12-well plates and were treated with the indicated drugs the following day. Fresh media and drug were added every 2 days. After 5–7 days cells were counted using a Z1 Coulter Particle Counter (Beckman Coulter).

### Western blots.

Cells were lysed in RIPA buffer (Thermo Fisher Scientific, PI89901) containing protease (Thermo Fisher Scientific, PI87786) and phosphatase inhibitor (MilliporeSigma P572, P0044) cocktails. Protein concentration was determined by BCA assay (Thermo Fisher Scientific). Quantified protein samples were separated by electrophoresis on 4%–20% ready-made Tris-Glycine gels (Invitrogen) and transferred to PVDF membranes (MilliporeSigma). Membranes were blocked with 5% skim milk for 1 hour and incubated overnight with 1 or more primary antibodies: phosphorylated histone H3 (Cell Signaling Technology, 3377S), α-tubulin (MilliporeSigma, DM1A), β-tubulin (Cell Signaling Technology, 2146S), and β-actin (MilliporeSigma, A1978). Western blot quantification was performed using densitometry analysis on ImageJ software (NIH).

### Intracellular tubulin polymerization assay.

We plated 50,000 cells per well in a 12-well plate, 24 hours before treatment with increasing concentrations of rigosertib with and without uric acid (MilliporeSigma U2625) in corresponding media. After drug treatment, the cells were lysed in a hypotonic lysis buffer (1 mM MgCl_2_, 2 mM EGTA, 20 mM Tris-HCl at pH 6.8, 0.15% IGEPAL, 5 μM paclitaxel) for 10 minutes at 37°C. The lysates were centrifuged at 21,000*g* for 10 minutes at room temperature. Equal volumes of the resulting supernatants (containing soluble tubulin) and pellet (containing microtubules) for each treatment condition were subjected to SDS-PAGE followed by immunoblotting for α-tubulin.

### Cell cycle and cell death analysis.

For cell cycle analysis cells were treated with 100 nM rigosertib overnight in corresponding media. The next day cells were washed with PBS, trypsinized, quenched, and washed 2 times with PBS. After centrifugation at 188*g*, cells were fixed with ethanol at 4°C. Fixed cells were vortexed for 20 minutes at 4°C, washed 2 times with PBS, and stained with propidium iodide (Thermo Fisher Scientific, AAJ66584AB). Stained cells were analyzed using CytoFLEX and Gallios flow cytometers (Beckman Coulter). Data were analyzed using FlowJo software. For the cell death assay cells were treated overnight with 200 nM rigosertib in corresponding media. Trypsinized cells were suspended in 300 μL FACS buffer and stained with propidium iodide for 30 minutes. Cells were analyzed using a CytoFLEX flow cytometer, and data were analyzed using FlowJo software.

### Drug screen.

The MAPS platform (38) was used to test both a commercial anticancer drug library and a custom-curated metabolic inhibitor drug library. Screen was performed at the ICCB-Longwood Screening Facility. SUM149 cells were seeded at a density of 500 cells per well in a final volume of 30 μL per well of 384-well plates. After 24 hours, a Seiko Compound Transfer Robot pin transferred 100 nL of each drug library into wells with plated cells. Following pin transfer, 20 μL of cell culture medium was added to all wells, resulting in each drug being applied at a final 10-point concentration series ranging from 20 μM to 1 nM. After 72 hours of drug treatment, the cells were washed with PBS, fixed with 4% formaldehyde, and stained with 5 mg/mL bisbenzimide. An Acumen Cellista plate cytometer was used to image plates and determine the cell numbers in individual wells. X,Y plots were generated comparing relative numbers of surviving RPMI and HPLM cells with concentrations of each drug tested. AUC values were calculated for each plot, and drugs were ranked based on the difference between the AUCs for RPMI and HPLM cells.

### LC-MS metabolite analysis.

LC-MS metabolite analysis was performed as previously described (71). Metabolites were extracted using 80% ice-cold methanol. A Vanquish UPLC system was coupled to a Q Exactive HF mass spectrometer equipped with heated electrospray ionization (ESI; Thermo Fisher Scientific). Chromatographic separation was performed with a SeQuant ZIC-pHILIC LC column, 5 μm, 150 × 4.6 mm (MilliporeSigma), with a SeQuant ZIC-pHILIC guard column, 20 × 4.6 mm (MilliporeSigma). Mobile phase A was 10 mM (NH_4_)_2_CO_3_ and 0.05% NH_4_OH in H_2_O, and mobile phase B was 100% acetonitrile. The column chamber temperature was set to 30°C. The mobile-phase gradient was as follows: 0–13 minutes: 80% to 20% of mobile phase B, 13–15 minutes: 20% of mobile phase B. ESI was performed in both positive and negative modes. The MS scan range was *m/z* 60–900. The mass resolution was 120,000 and the automatic gain control target was 3 × 10^6^. The capillary voltage was 3.5 kV and the capillary temperature was 320°C. A total of 5 μL of sample was loaded. LC-MS peaks were manually identified and integrated with EL-Maven (Elucidata) by matching with an in-house library. MetaboAnalyst was used to normalize the peak areas of target metabolites to the median fold-change across all identified metabolites, calculate fold-changes, and calculate *P* values.

### Gas chromatography–MS metabolite analysis.

Polar metabolites were prepared for analysis by first drying the samples in individual microcentrifuge tubes, then adding 15 μL of methoxyamine–hydrogen chloride in pyridine (Thermo Fisher Scientific) and incubating at 40°C for 90 minutes. The samples were then further incubated with 20 μL of *N*-(*tert*-butyldimethylsilyl)-*N*-methyl-trifluoroacetamide with 1% *tert*-Butyldimethylchlorosilane (MilliporeSigma) at 60°C for 60 minutes. The resulting derivatized solution was vortexed briefly, centrifuged, and transferred into polypropylene gas chromatography–MS (GC-MS) vials (Agilent). Subsequent metabolite abundance analysis was conducted using an Agilent 6890N GC coupled with 5975B Inert XL MS. An Agilent J&W DB-35 ms column was used. Chromatography-grade helium (Airgas) was used as the carrier gas, flowing at a rate of 1 mL/min. Depending on sample abundances, either 1 or 2 μL of samples was injected using either split or splitless modes. The 6890N GC inlet temperature was set to 270°C, and the oven temperature was initially set to 100°C, then raised to 300°C at a rate of 2.5°C/min. Electron impact ionization mode with 70 eV was used for 5975B MS measurement. Acquisition was performed using scan mode with a detection range of *m/z* 150–625. Mass isotopomer distributions were corrected for natural isotope abundance. Detailed methods are published (72).

### MD simulations.

The starting points for our simulations were the Protein Data Bank structures for colchicine- and rigosertib-bound tubulin (1SA0, ref. 73; and OV7, ref. 47, respectively). We used a single tubulin dimer from each structure, removing the additional proteins that were added to promote crystallization. As a control, we also removed colchicine from the 1SA0 structure to create apo-tubulin to be used as a reference structure. Each complex had GTP in α-tubulin and GDP in β-tubulin, and we used CGenFF (74) to create initial force field parameters for both colchicine and rigosertib. Each system was then solvated using TIP3 water with Na^+^ and Cl^–^ added to both neutralize the system charge and set the ionic strength to 50 mM. Simulations were carried out using NAMD (75) using the CHARMM36 (76) force field. Following heating we performed 0.5 μs equilibration simulations to remove the effects of the stathmin in the crystal structures. We then performed 4 independent 0.5 μs simulations of each structure at 300°K in an NpT ensemble with 1 atm pressure. Bonded hydrogens were fixed to allow us to use 2 fs time steps. We employed Particle Mesh Ewald (77) for long-range electrostatics and used a 10 Å cut-off and 8.5 Å switch distance for van der Waals interactions. This resulted in more than 2 μs of simulation data for each of the apo, colchicine, and rigosertib systems. Analysis was done using bio3D (78), and images were created using VMD (79).

### Molecular docking studies.

For uric acid docking studies, we used Maestro (Schrödinger). We took 40 different structures from the rigosertib and colchicine simulations (20 from each) for docking of uric acid. The following workflow was used for each structure: 1) the ligand (uric acid) and protein (tubulin-drug complexes) were prepared to be compatible with Maestro applications using LigPrep and ProteinPrep, respectively; 2) we generated possible binding sites on the tubulin complex using SiteMap; 3) we created receptor grids to be used for docking via Glide; and 4) we docked the prepped ligand (uric acid) to the tubulin complex using ligand docking by Glide. To evaluate the binding free energy of rigosertib with and without uric acid, we utilized the Molecular Mechanics Generalized Born Surface Area methods in Prime.

### CETSA.

K562 cells were treated with 40 μM rigosertib for 4 hours in the corresponding media, after which cells were washed with 1× PBS. Next, cells were resuspended in PBS containing 1× Halt protease inhibitor cocktail (Thermo Fisher Scientific, PI87786) and counted using a Z1 Coulter Particle Counter (Beckman Coulter). A total of 700,000 cells were dispensed in PCR tubes and heated at the indicated temperatures for 3 minutes in a thermocycler (Bio-Rad). After heating, cells were cooled to 20°C and lysed by 3 cycles of freeze/thaw in liquid nitrogen. Following lysis, denatured proteins were separated by centrifugation at 21,000*g* for 10 minutes at 4°C. The lysate was dissolved in 6× loading buffer and run on SDS-PAGE as described in the *Western blots* section.

### Statistics.

Statistical analyses were performed using GraphPad Prism 9 and Microsoft Excel. Unpaired 2-tailed *t* tests were used in most experiments. Where applicable 1-way ANOVA followed by Tukey’s multiple comparisons test and 2-way ANOVA were used. *P* < 0.05 was considered statistically significant.

### Study approval.

This study was approved by the Institutional Biosafety Committee at the University of Illinois at Chicago.

### Data availability.

Underlying data are available in the Supporting Data Values XLS file.

## Author contributions

JLC and VR conceived the study; JLC, VR, PP, MEO, PD, PGS, ISH, and DS developed methodology; JLC, VR, PP, AT, PD, and MEO performed validation; JLC, VR, PP, PAB, KOC, and MEO performed formal analysis; JLC, VR, PP, MEO, AT, JEE, PD, PAB, KOC, CS, YH, AN, PGS, GMD, ISH, DS, FMM, GS, and WD performed investigation; JLC, VR, ISH, and DS provided resources; JLC and VR performed data curation; JLC, VR, MEO, PP, and PD wrote the original draft; JLC, VR, PP, MEO, and PD performed review and editing; JLC, VR, PP, and MEO performed visualization; and JLC supervised, administered the project, and acquired funding.

## Supplementary Material

Supplemental data

Unedited blot and gel images

Supplemental table 1

Supplemental video 1

Supplemental video 2

Supporting data values

## Version 1 05/30/2024

In-Press Preview

## Version 2 07/08/2024

Electronic publication
