# Supplementary material for: Drug screening in human physiologic medium identifies uric acid as an inhibitor of rigosertib efficacy
Source: JCI Insight. 2024 May 30;9(13):e174329. doi: 10.1172/jci.insight.174329 (PMC11383364; doi:10.1172/jci.insight.174329)
Supplement: Supplemental data [file jciinsight-9-174329-s022.pdf]

Supplemental figures

Fig s1.

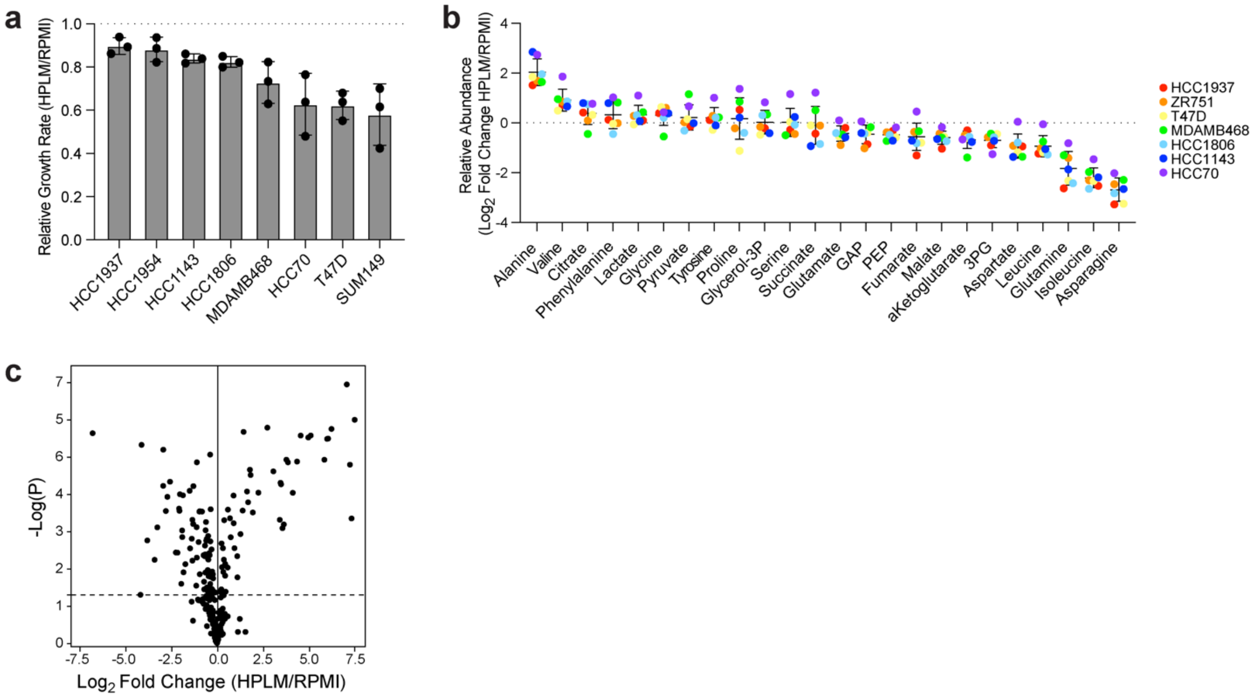

Supplementary Figure 1. Growth of breast cancer cells in HPLM causes remodeling of intracellular metabolite abundance. Related to Figure 1.

(a) Relative growth rate of breast cancer cell lines cultured in HPLM vs RPMI. Data is represented as mean ± SD from triplicate samples. (b) GC-MS analysis of relative metabolite abundance in breast cancer cells growing in HPLM vs RPMI. Data is represented as the mean of triplicate samples for each cell line. (c) LC-MS analysis of relative metabolite abundance in HCC1806 cells growing in HPLM vs RPMI. Data is represented as the mean of triplicate samples.

Fig s2.

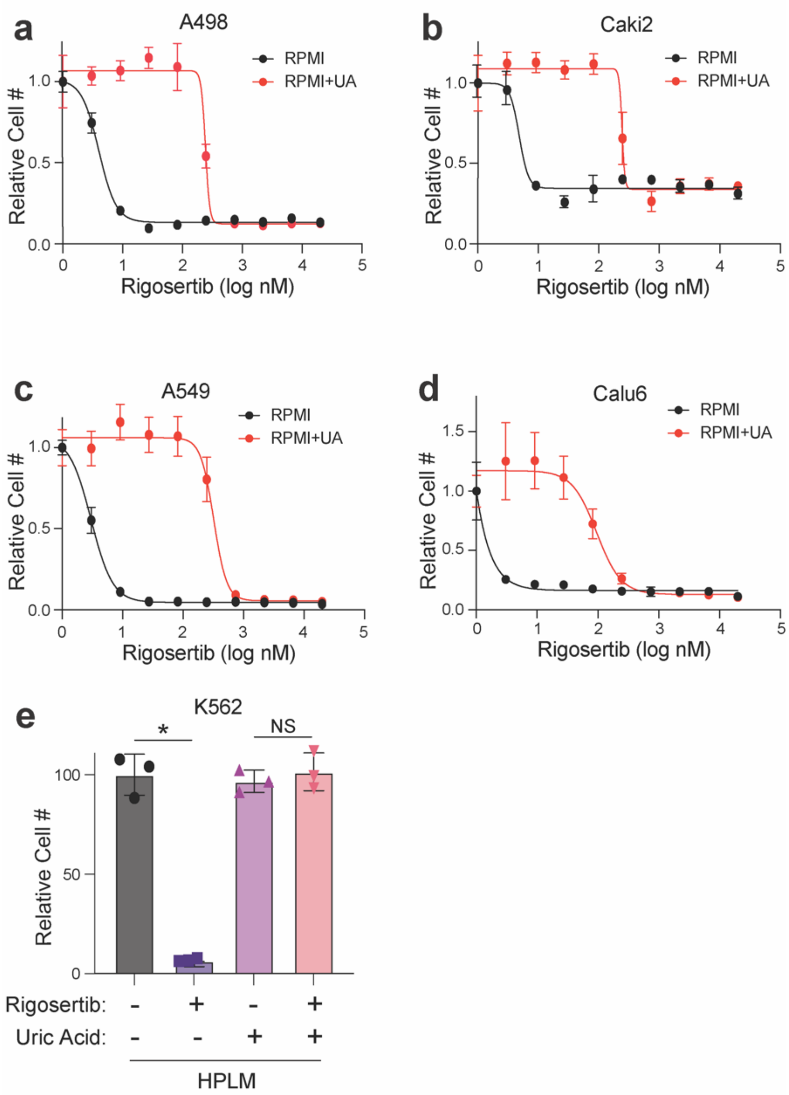

Supplementary Figure 2. Uric acid reduces sensitivity to rigosertib in diverse cell lines. Related to Fig 3.
(a – d) Dose response curves of A498 (a), Caki2 (b), A549 (c), and Calu6 (d) cells treated with rigosertib in RPMI vs RPMI + uric acid. (e) Growth of K562 cells treated with 100 nM rigosertib in HPLM – UA vs HPLM. For all panels, data is represented as mean ± SD of triplicate samples. * indicates $p < 0.05$ from unpaired two-tailed t-test. NS (not significant) indicates $p > 0.05$.

Fig s3.

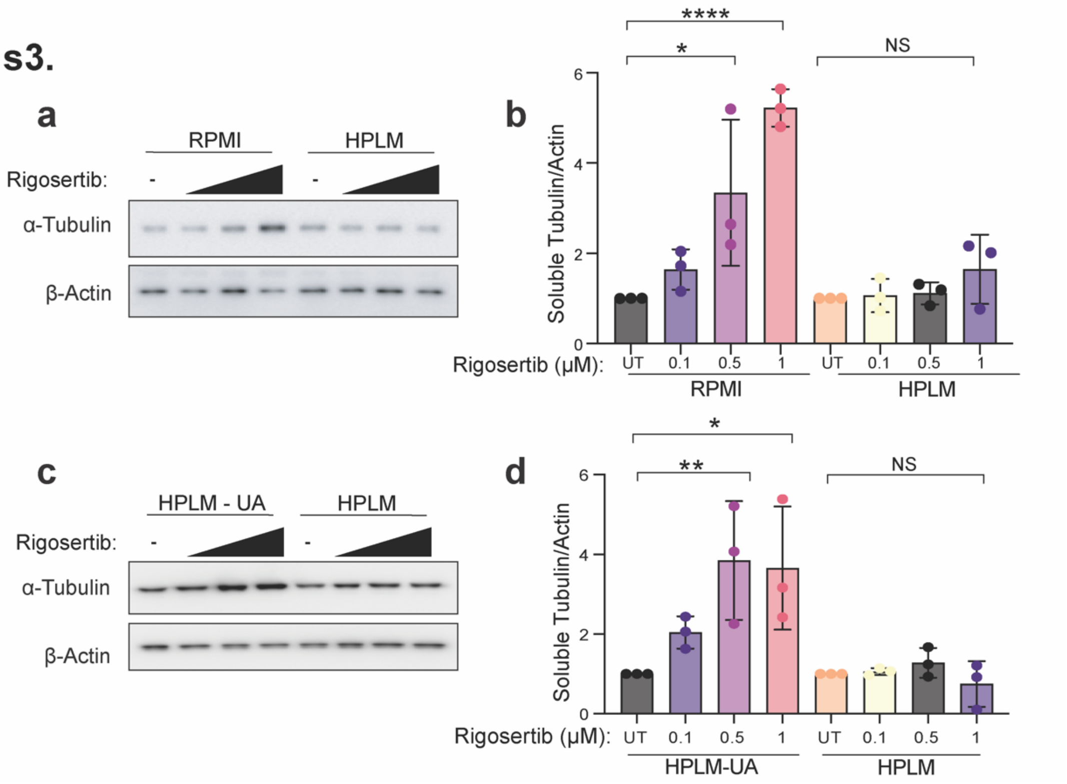

Supplementary Figure 3. Uric acid inhibits the microtubule destabilizing activity rigosertib. Related to fig 4.
(a) Western blot of soluble α-tubulin from HCC1806 treated with increasing doses of rigosertib (0.1 μM, 0.5 μM and 1 μM) for 4 hr in RPMI and HPLM. (b) Quantification of western blots from (a). Data is represented as mean ± SD from three independent experiments. **** $p < 0.0001$, ** $p < 0.01$, * indicates $p < 0.05$ from one way ANOVA followed by Tukey's multiple comparison test. NS (not significant) indicates $p > 0.05$. (c) Western blot of soluble α-tubulin from HCC1806 treated with increasing doses of rigosertib (0.1 μM, 0.5 μM and 1 μM) for 4 hr in HPLM and HPLM – UA. (d) Quantification of western blots from (c). Data is represented as mean ± SD from three independent experiments. * indicates $p < 0.05$ from one way ANOVA followed by Tukey's multiple comparison test. NS (not significant) indicates $p > 0.05$.

Fig s4.

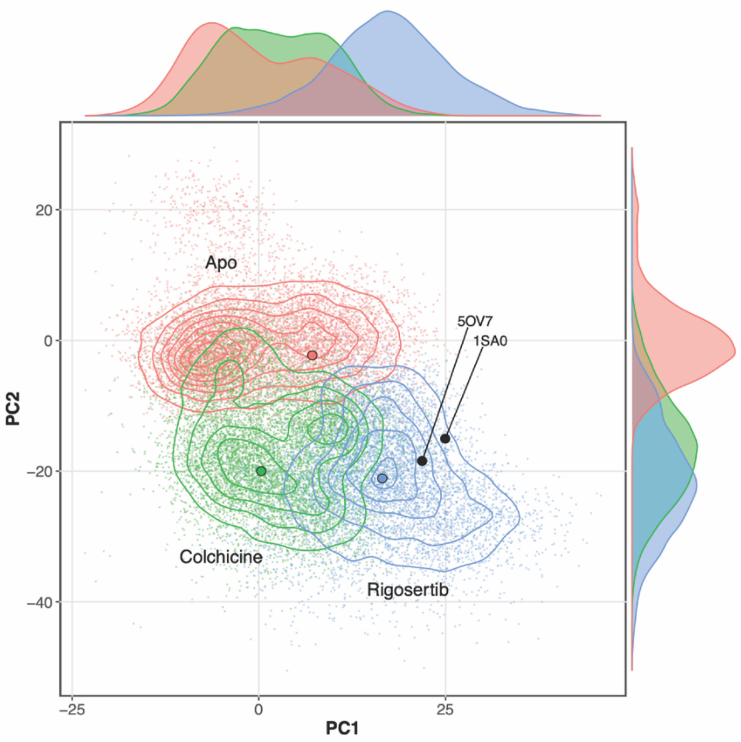

Supplementary Figure 4. Rigosertib and colchicine have overlapping effects on tubulin structure.
Comparison of apo (red), colchicine-bound (green), and rigosertib-bound tubulin (blue) using PCA analysis. Crystal structures of rigosertib (5OV7) and colchicine (1SA0) are superimposed on the PCA space, highlighting some of the limited information in the crystal structures. Both colchicine and rigosertib cause a conformational change that makes the dimer non-polymerizable (PC2), but rigosertib induces an additional conformational change that is distinct from colchicine (PC1).
