# Supplementary material for: Drug screening in human physiologic medium identifies uric acid as an inhibitor of rigosertib efficacy
Source: JCI Insight. 2024 May 30;9(13):e174329. doi: 10.1172/jci.insight.174329 (PMC11383364; doi:10.1172/jci.insight.174329)
Supplement: Unedited blot and gel images [file jciinsight-9-174329-s023.pdf]

Full unedited gel for Figure 2f

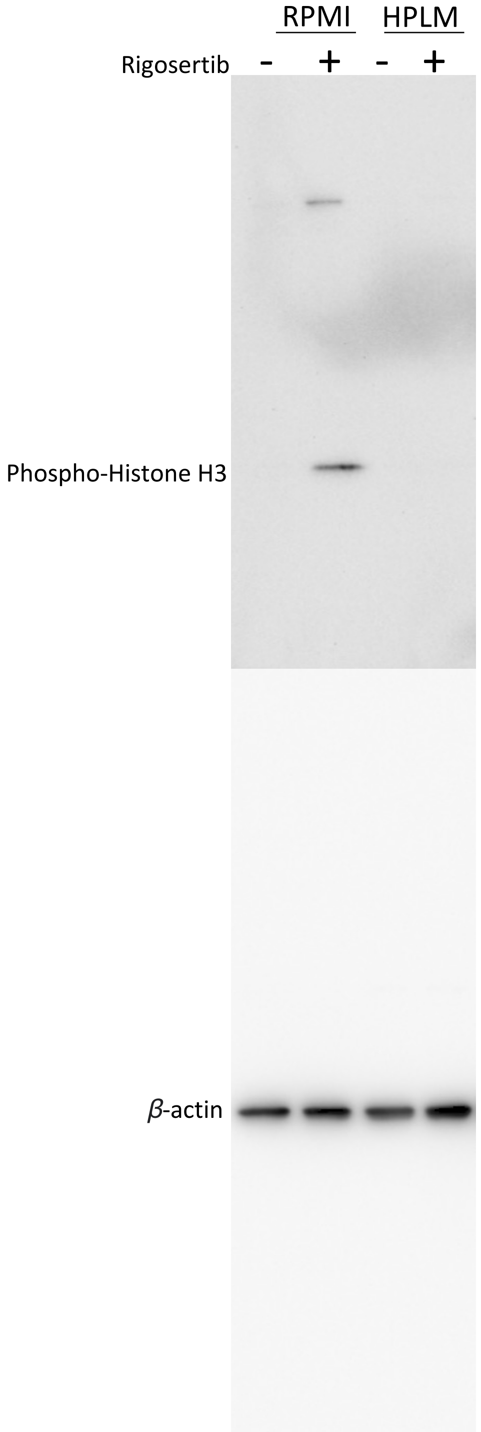

Antibody used

phospho-Histone H3 (Cell Signaling, 3377S)

β-actin-(Sigma, A1978)

Full unedited gel for Figure 3h

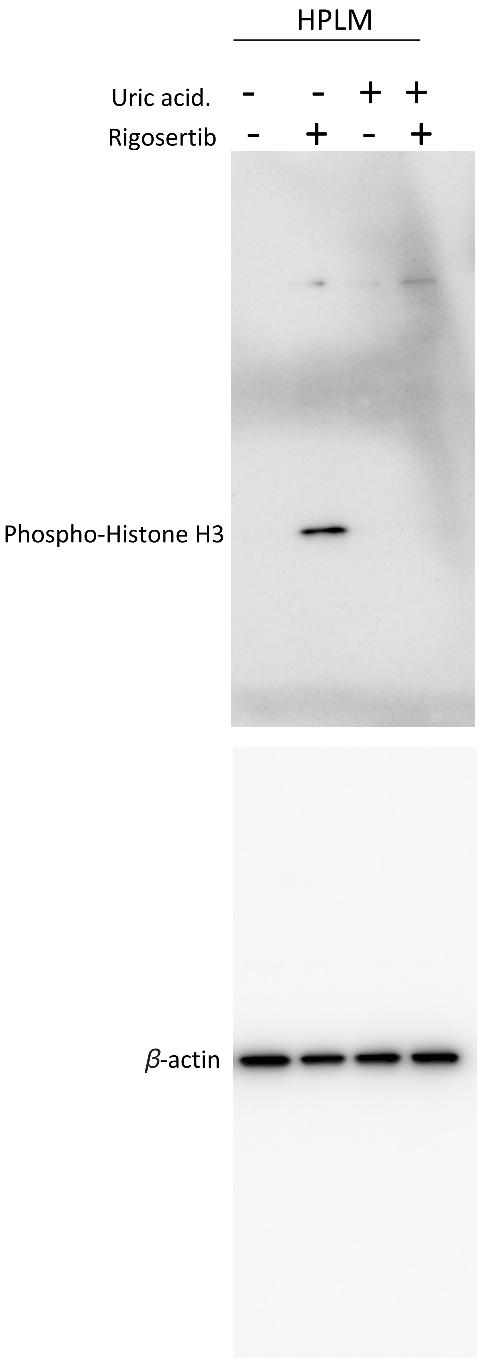

Antibody used:

phospho-Histone H3 (Cell Signaling, 3377S

β-actin-(Sigma, A1978)

Full unedited gel for Figure 4a

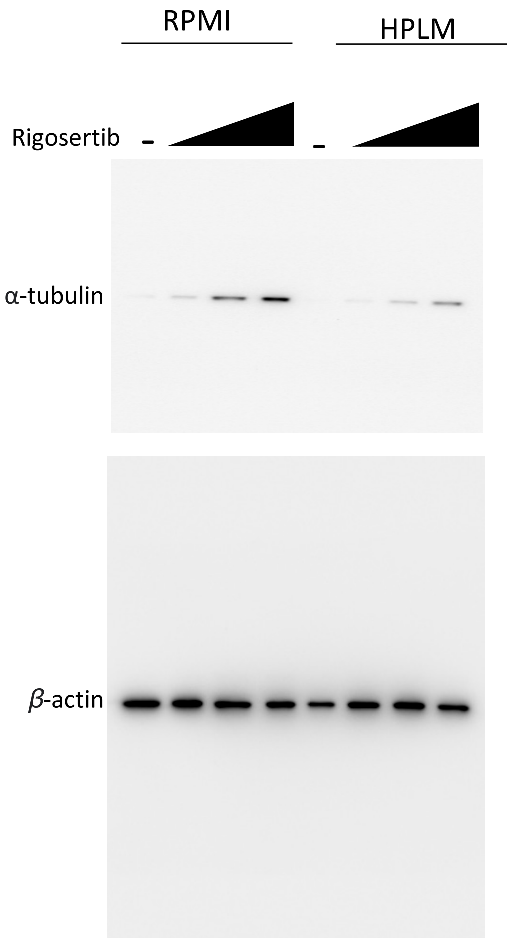

Antibody used:

α-tubulin (Sigma, T9026)
β-actin-(Sigma, A1978)

Full unedited gel for Figure 4c

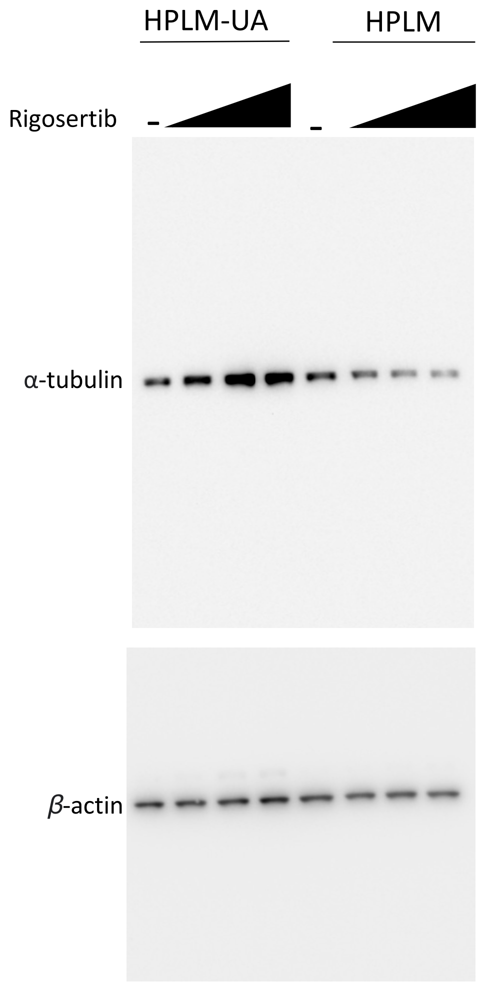

Antibody used:

α-tubulin (Sigma, T9026)
β-actin-(Sigma, A1978)

Full unedited gel for Figure 4i

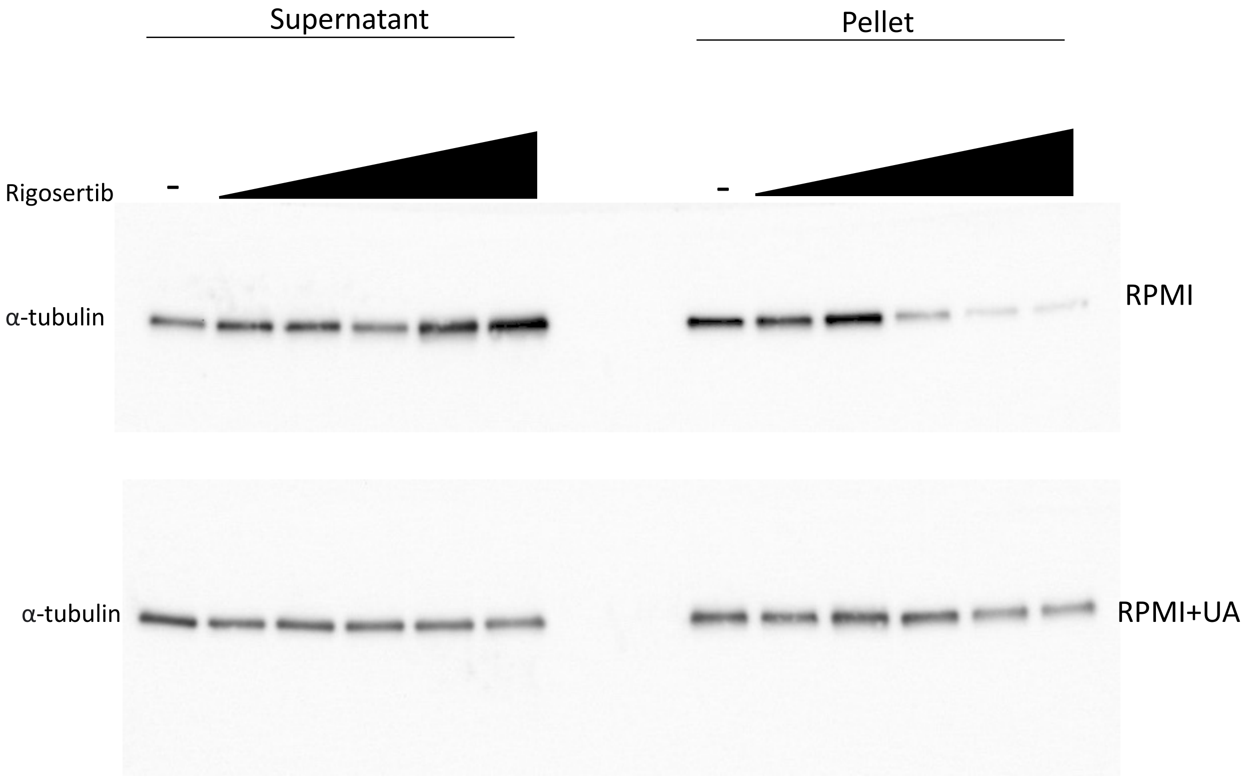

Antibody used:

α-tubulin (Sigma, T9026)

Full unedited gel for Figure 5d

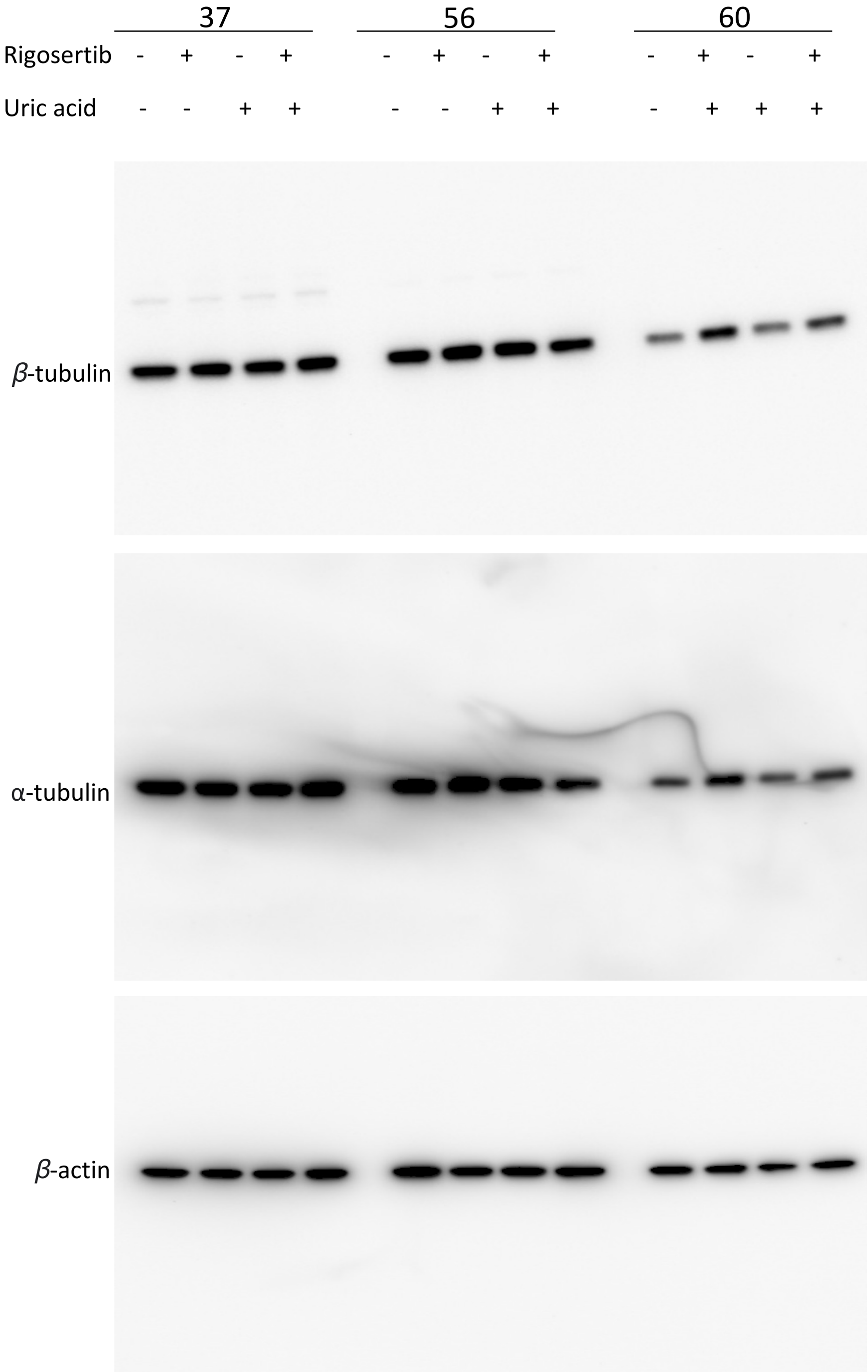

Antibody used:

α-tubulin (Sigma, T9026)
β-tubulin (Cell Signaling, 2146S)
β-actin-(Sigma, A1978)

Full unedited gel for Figure S3a

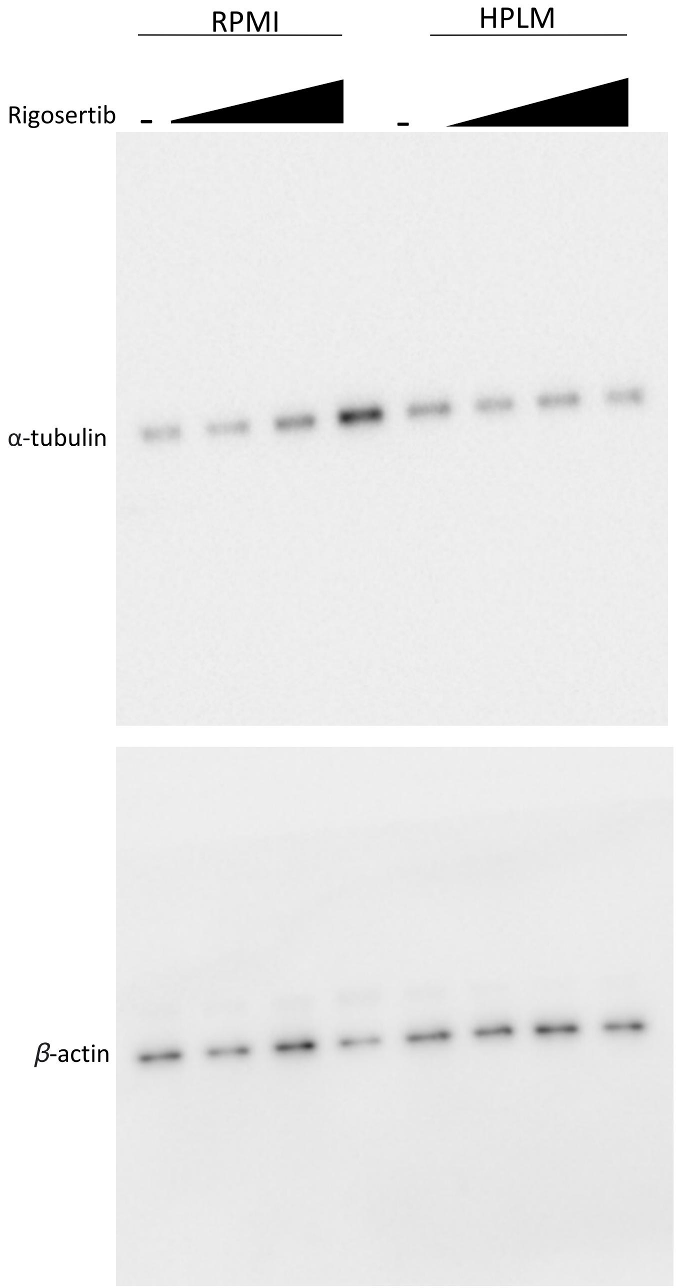

Antibody used:

α-tubulin (Sigma, T9026)
β-actin-(Sigma, A1978)

Full unedited gel for Figure S3c

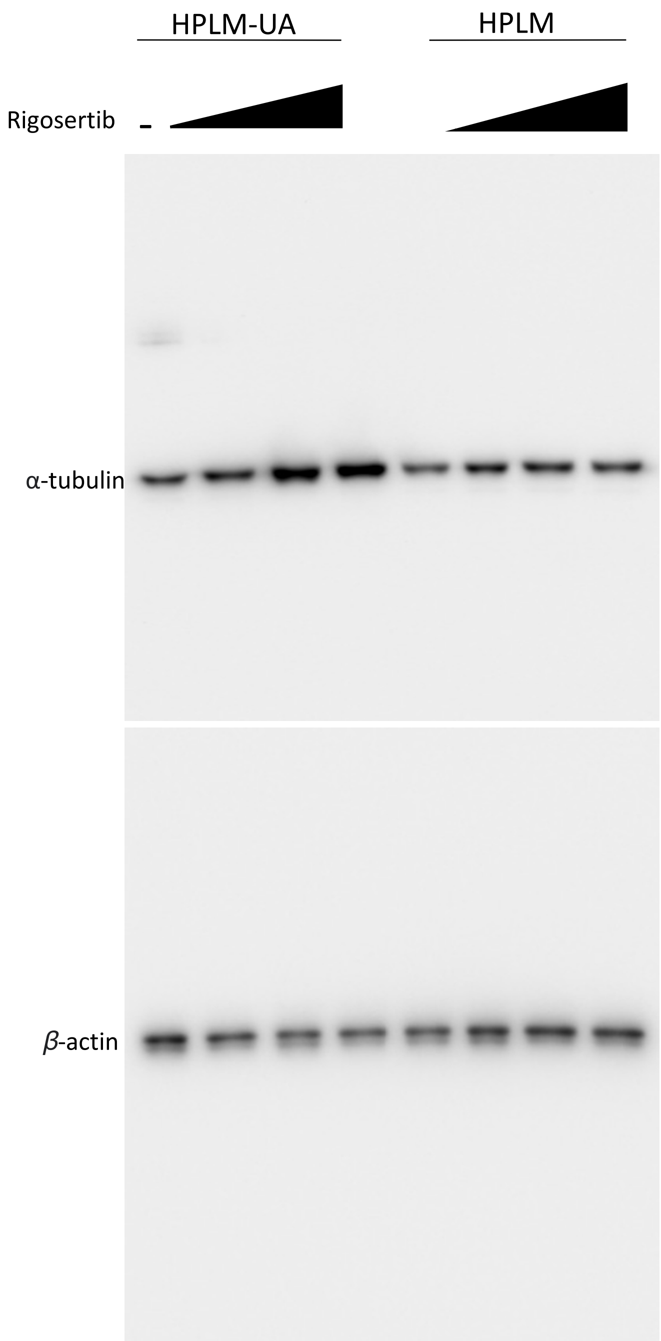

Antibody used:

α-tubulin (Sigma, T9026)
β-actin-(Sigma, A1978)
